# Isolation and encapsulation of bacteriophage with chitosan nanoparticles for biocontrol of multidrug-resistant methicillin-resistant *Staphylococcus aureus* isolated from broiler poultry farms

**DOI:** 10.1038/s41598-024-55114-5

**Published:** 2024-02-26

**Authors:** Mona M. Elsayed, Rasha M. Elkenany, Ayman Y. EL-Khateeb, Nehal M. Nabil, Maram M. Tawakol, Heba M. Hassan

**Affiliations:** 1 Department of Hygiene and Zoonoses, Faculty of Veterinary Medicine, Mansoura University, Mansoura, 35516 Egypt; 2 Department of Bacteriology, Immunology, and Mycology, Faculty of Veterinary Medicine, Mansoura University, Mansoura, 35516 Egypt; 3 Department of Agricultural Chemistry, Faculty of Agriculture, Mansoura University, Mansoura, 35516 Egypt; 4 Reference Laboratory for Veterinary Quality Control on Poultry Production, Animal Health Research Institute (AHRI), Agricultural Research Center (ARC), Nadi El-Seid Street, Dokki, 12618 Giza Egypt

**Keywords:** Broiler chicken farms, Chitosan nanoparticles, Encapsulation, MRSA, Phage, Biotechnology, Microbiology

## Abstract

This study was divided into two parts. The first part, the determination of methicillin-resistant *Staphylococcus aureus* (MRSA) prevalence in 25 broiler chicken farms, with the detection of multidrug resistant MRSA strains. The prevalence of MRSA was 31.8% (159 out of 500 samples) at the level of birds and it was 27% (27 out of 100) in the environmental samples. The highest antimicrobial resistance of the recovered MRSA strains was recorded to streptomycin (96%). All isolates (100%) had multidrug resistance (MDR) to four or more antibiotics with 16 distinct antibiotic resistant patterns, and multiple antibiotic resistance index (MARI) of 0.4–1. The second part, implementing novel biocontrol method for the isolated multidrug resistant MRSA strains through the isolation of its specific phage and detection of its survival rate at different pH and temperature degrees and lytic activity with and without encapsulation by chitosan nanoparticles (CS-NPs). Encapsulated and non-encapsulated MRSA phages were characterized using transmission electron microscope (TEM). Encapsulation of MRSA phage with CS-NPs increasing its lytic activity and its resistance to adverse conditions from pH and temperature. The findings of this study suggested that CS-NPs act as a protective barrier for MRSA phage for the control of multidrug resistant MRSA in broiler chicken farms.

## Introduction

Methicillin-resistant *Staphylococcus aureus* is a pathogen that has a negative impact on veterinary and public health sectors. MRSA has been isolated from human and animals including broiler chicken^1–3^. MRSA has been found to harbor resistance towards different classes of antibiotics. Global public health is facing a major issue with the rise in antibiotic resistance rates for drugs that are sold commercially^4^. The high phenotypic resistance is mainly attributed to the misuse of antibiotics in both developed and developing countries^5^. Therefore, the control of antibiotics used during production cycles must be urgently implemented. The new and potential alternative to antibiotics against MRSA is the use of bacteriophages^4^. The ability of bacteriophages to destroy pathogenic bacteria, host specificity, clinical safety, self-replicating nature and easy and low-cost isolation make bacteriophages ideal and safe^5^. Meanwhile, there are limits for the application of phages as therapeutic agents, notably instability of lytic phages at low pH and high temperature which affect phage viability and reduce their effectiveness^6^. These factors must be considered for effective utilization of bacteriophages as biocontrol agents^7^. Bacteriophage encapsulation with nanoparticles protect it aganist adverse environmental conditions^8^. Previous studies have revealed the possibility of phage microencapsulation of bacteriophages with using chitosan as biomaterials^9,10^ which related to its solubility in acidic pH in addition to the biocompatible and biodegradable properties^11^. Antimicrobial properties of CS-NPs allow a synergistic antimicrobial effect against various pathogenic agents^5^. The process of encapsulation could protect bacteriophages against adverse environments such as acidity and improve its effectiveness^12^. The epidemiological studies on MRSA in animals and the appropriate control methods for it are not sufficient. Therefore, this study was divided into 2 parts. The first part, including the determination of MRSA prevalence in broiler chickens and farm environment. The second part, isolation of specific multidrug resistant MRSA phage and detection of its survival rate at different pH and temperature degrees and lytic activity in the free and encapsulated form with CS-NPs.

## Methods

### Collection of samples

This study was performed by following the animal ethics guidelines and approved by Medical Research Ethics Committee of Mansoura University with code number; MU-ACUC (VM.R.23.08.118). All experiments were performed in accordance with relevant guidelines and regulations. Also, all methods are reported in accordance with ARRIVE guidelines.

Twenty-five broiler farms in the El-Dakahlia and Damietta governorates, Egypt were selected on the bases the cooperation of the farm’s owners during sampling. Six hundred samples were collected aseptically from 25 broiler farms. These samples were divided into bird samples (n = 500) and environmental samples (n = 100). The bird samples include cloacal swabs and internal organs (heart, lung and liver). One hundred and twenty-five cloacal swabs (5 birds from each farm) were collected from apparently healthy birds which released in the farm, meanwhile the internal organs were collected from diseased birds (125 sample for each type; 5 birds from each farm) after they were euthanized by head dislocation. Meanwhile, the environmental samples include litter, water, feed samples and workers hand swaps (25 composting samples for each; one composite sample for each farm), informed consent was obtained from all workers from which swaps samples were taken. Samples were transported aseptically in an icebox to the laboratory for further investigations.

### Isolation and identification of methicillin-resistant *Staphylococcus aureus* (MRSA)

Samples were enriched in 5 ml tryptone soy broth (TSB, Lab M Limited, Lancashire, UK) containing 6.5% NaCl overnight before culture on CHROM agar MRSA medium (CHROM agar, France) and incubated at 37 °C for 48 h according to Diederen et al.^13^. Pink to mauve colonies were purified on mannitol salt agar to obtain pure colonies. Suspected *S. aureus* colonies were identified by Gram’s stain, hemolytic pattern and coagulase test according to ISO 688-1^14^.

The resistance of *Staphylococcus aureus* isolates was screened in vitro for cefoxitin by the disk diffusion method according to Clinical and Laboratory Standards Institute^15^ on Mueller–Hinton agar (Oxoid Ltd., Hampshire, England) for MRSA identification^16^.

### Molecular detection and characterization of the MRSA isolates

Extraction and purification of the phenotypical MRSA DNA were performed and screened for the presence of *mec A* and *fem A* genes^17^ by multiplex PCR. The following specific primers: F-5′ GTA GAA ATG ACT GAA CGT CCG ATA A3′and R-5 ′CCA ATT CCA CAT TGT TTC GGTCTA A3′ for *mec A* gene (310 bp) and F-5′-AAA AAA GCA CAT AAC AAG CG-3′and R-5′GATAAA GAA GAA ACGAGC AG-3′ for *fem A* gene (132 bp) according to Johnson et al.^18^. Each PCR reaction mixture was prepared from 10X reaction buffer (5 μL), template DNA (5 μL), each primer (1 μL), 10 mM dNTP mixture (3 μL), and Taq polymerase (1 μL) and the volume was completed to 50 μL with nuclease-free deionized water. Amplification was performed into Tianlong PCR thermocycler (initial denaturation at 94 °C for 6 min denaturation at 94 °C for 45 s, annealing at 55 °C for 30 s and extension at 72 °C for 45 s, and with final extension at 72 °C for 6 min). Finally, gel electrophoresis for PCR products on 1.5% agarose gel containing ethidium bromide (0.5 g/mL) was performed in a horizontal system for 55 min.

### Detection of multidrug resistant MRSA

Fifty MRSA strains were selected (2 isolates from each farm) for detection of multidrug resistant MRSA strains against eleven different antibiotic discs (representative of the common antibiotics used in poultry settings) including oxacillin (1 μg), vancomycin (30 μg), penicillin G (10 μg), oxytetracycline (30 μg), streptomycin (25 μg), trimethoprim (5 μg), amoxicillin/clavulanic acid (30 μg), ampicillin (10 μg), nalidixic acid (30 μg), sulfamethoxazole/trimethoprim (300 μg), and kanamycin (30 μg) (Oxoid, Hampshire, England). The inhibition zones diameter was measured then interpreted according to the interpretation table of the Clinical and Laboratory Standard Institute^19^.

### Isolation of phages against multidrug resistant MRSA

A total of 15 wastewater samples were collected from 5 poultry slaughterhouses located at Dakahlia Governorate, Egypt. Fifty ml of each sample were filtered using 0.22 μm membrane filter then mixed with 50 ml Luria Bertani (LB) broth (Oxoid, USA) containing multidrug resistant MRSA (10^8^ CFU/ml). The mixture was incubated overnight at 37 °C then was centrifuged at 8000×*g* for 10 min. The prepared filtrate was examined for the detection of lytic bacteriophage by double layer agar method. Briefly, 0.1 ml of overnight broth culture of multidrug resistant MRSA isolate was mixed with soft nutrient agar (0.7%) and then poured onto solid LB agar plates. Ten μL of the prepared phage suspension was added to the bacteria on the double layer agar plates and incubated at 37 °C/overnight^20^. Plaque assay was performed to purify the prepared *S. aureus* phage using double layer method^21,22^.

### Electron microscope characterization of isolated phage

The morphological characters of the isolated bacteriophage were detected by electron microscope (Hitachi H600A) in Faculty of Agriculture, Mansoura University, Egypt. According to Abdel-Haliem and Askora^23^, the purified bacteriophage was stained using uranyl acetate and a drop of the bacteriophage suspension (10^10^ PFU/ml) was placed on 200 mesh copper grids with carbon-coat formvar films. The bacteriophages were examined, named and classified.

### One-step growth curve

The one-step growth experiment was performed for MRSA phage^24^. The Multidrug resistant MRSA was diluted in 1 mL of BHI broth until an OD_600_ of 0.5 (1.5 × 10^8^ CFU). 0.9 mL of BHI broth mixed with 0.1 mL of isolated MRSA phage solution (1 × 10^8^ PFU/mL). After 5 min, the mixture was centrifuged (13,000×*g* for 1 min) to discard free phage particles. The sediments (the phage-infected bacterial cells) were diluted in MS broth and were incubated at 30 °C. At intervals of 5 min, samples were collected for the detection of phage titers by the double-layer agar method and calculation of the burst size of phages. This experiment was performed in triplicate.

### Chitosan nanoparticles (CS‑NPs) preparation and phage-CS-NPs Encapsulation

Chitosan nanoparticles were prepared with aqueous sodium tripolyphosphate (STPP) solution using the ion gelation method. Chitosan, deacylated chitin, poly(D-glucosamine) of medium molecular weight from shrimp shells particle grade (deacetylation degree, min 90% and viscosity, 200–800 cps), Sigma-Aldrich Com, GmbH. 500 mg of chitosan was dissolved in an aqueous solution of acetic acid (1%, w/v), to make a 5 mg/mL concentration until being homogenous by magnetic stirrer (150 rpm) at 25 °C for 4 h till complete solubility according to^12^. The crosslinking of chitosan with aqueous solution of STPP (0.67%, w/v) at ratio of (1:1 v/v) was applied with dropwise addition of STPP at constant flow rate of 10 drops per minute to 100 mL of the chitosan solution and the stirring at 500 rpm was continued for more 2 h to allow anionic molecules diffuse into the mixture of positively charge chitosan molecules and crosslinking occurs leading to nanoparticles formation according to Nalini et al.^25^. For encapsulation of the isolated phage with CS-NPs, 0.1 mL of the phage solution (63 × 10^10^ PFU/mL) was added to CS-NPs solution^12^ with continuous stirring for more 2 h to allow encapsulation process according to Chen et al*.*^26^.

### Transmission Electron Microscope (TEM) of chitosan nanoparticles (CS-NPs)

Transmission Electron Microscope (TEM) analysis was used for characterization of nature, size, morphology and crystallography nature of the prepared chitosan nanoparticles (CS-NPs)^27^.

#### Stability of encapsulated and free bacteriophages

The stability of free isolated bacteriophage and encapsulated bacteriophages was investigated in various temperatures and PH, the double-layer agar method was conducted to count plaque formation using phage solutions incubated at various temperatures (4, 25, 40 and 60 °C)^28^ and various PH values (1.5, 2.5, 3, 4, 7, 8, 9 and 12) using NaOH or HCl. The 0.2 ml of phage suspension in SM buffer (10^10^PFU/ml) was incubated at various temperatures. After 1 and 2 h the phage suspension was subsequently diluted serially with SM buffer, and 0.1 ml of diluted suspension was mixed with 0.1 ml of host strain (5 × 10^8^ CFU/ml). The mixture was added to 3 ml of soft agar to generate a bacterial lawn on TSA plates, and the number of plaques was counted.

### In-vitro Lytic activity of encapsulated bacteriophages against multi-drug resistant MRSA

The lytic activity of free bacteriophage, encapsulated bacteriophage and chitosan nanoparticles alone against multi-drug resistant MRSA isolate were tested in vitro. The lytic activity was detected and represented in a time-kill curve. The experiment was performed in microtitration plate (24-well). 1.8 mL of multidrug resistant MRSA suspension (8.0 × 10^6^ CFU/mL) was distributed in each well^29^. Encapsulated bacteriophage (200 µL) was added to all wells at which the final concentration was 10^5^–10^7^ PFU/mL. Negative control was represented in sterile BHI broth (0.2 mL). At zero time, the multi-drug resistant MRSA strain and phage titers were confirmed. The plates were incubated at 30 °C, and the OD600 was read every 2 h for 24 h. The experiment was performed in triplicate^30^.

### Statistical analysis

Data was recorded in Microsoft Excel (version 15.0), and statistical analysis was carried out using Statistical Package for the Social Sciences software version 22.

### Ethics approval

This protocol was performed by following the animal ethics guidelines and approved by Medical Research Ethics Committee of Mansoura University MU-ACUC (VM.R.23.08.118). Also, all methods are reported in accordance with ARRIVE guidelines.

## Results

### Prevalence of MRSA in the examined bird and environmental samples

The prevalence of MRSA was screened in this study in 500 bird samples including 500 cloacal swabs, heart, lung and liver samples in addition to 100 environmental samples. At the level of birds, out of 500 samples, MRSA were detected in 159 (31.8%) samples (Table 1) including 62, 42, 33 and 22 strains from the examined cloacal swabs, heart, lung and liver samples, respectively. In environmental samples, MRSA was 27% (27 out of 100) which was most commonly in litter and water (48% and 28%, respectively) followed by worker hand swaps (20%) and feed (12%).

**Table 1:** Prevalence of MRSA in broiler chicken farms on the level of birds and environmental samples.

Sample type	Total no. of samples	MRSA-positive samples (%)
Bird		
Cloacal swabs	125	62 (49.6)
Heart	125	42 (33.6)
Lung	125	33 (26.4)
Liver	125	22 (17.6)
Total	500	159 (31.8)
Environmental		
Litter	25	12 (48)
Water	25	7 (28)
Feed	25	3 (12)
Workers hand swaps	25	5 (20)
Total	100	27 (27)

### Antimicrobial resistance of MRSA

The antimicrobial susceptibility and resistance of the selected 50 isolates were detected against 10 antibiotics which revealed a highest resistance to Streptomycin (S) (96%) followed by penicillin G (P) and sulfamethoxazole/trimethoprim (SXT) (72% for each), Oxytetracycline (OTC) and Nalidixic acid (70%), and amoxicillin/clavulanic acid (AMC), ampicillin (AMP) (52% for each). Meanwhile, kanamycin (K) and vancomycin (VA) showed a lower rate of resistance (21% and 19%, respectively) (Table 2). In addition, all isolates (100%) had multidrug resistance (MDR) to four or more antibiotics, MARI of 0.4–1. The examined MRSA strains demonstrated 16 different antibiotic resistant patterns (Table 3), revealed the high prevalence of MDR among the isolated MRSA strains.

**Table 2:** Antibiotics Susceptibility pattern of the isolated *MRSA strains.*

Antibiotics	MRSA (50 isolates)		
	No of sensitive isolates (%)	No of Intermediate Isolates (%)	No of resistant isolates (%)
OXA	7 (14)	12 (24)	31 (62)
VA	31 (62)	10 (20)	9 (18)
P	6 (12)	8 (16)	36 (72)
OTC	6 (12)	9 (18)	35 (70)
S	0	2 (4)	48 (96)
SXT	2 (4)	12 (24)	36 (72)
AMC	9 (18)	15 (30)	26 (52)
AMP	13 (26)	11 (22)	26 (52)
NA	3 (6)	12 (24)	35 (70)
K	4 (8)	15 (30)	21 (42)

*OXA* oxacillin, *VA* vancomycin, *P* penicillin G, *OTC* oxytetracycline, *S* streptomycin, *SXT* sulphamethazole/trimethoprim, *AMC* amoxicillin/clavulanic acid, *AMP* ampicillin, *NA* nalidixic acid, *K* kanamycin.

**Table 3:** Antimicrobial resistance profiles of isolated *MRSA*.

Antibiotics pattern profile	Antibiotics	No. of resistance antibiotics	No. of isolates (isolates ID)	MARI
1	OXA, VA, P, OTC, S, SXT, AMC, AMP, NA, K	10	4 (1, 8, 18, 33)	1
2	OXA, VA, P, OTC, S, SXT, AMC, AMP, NA	9	4 (3, 19, 24, 42)	0.9
3	OXA, VA, P, OTC, S, SXT, AMC, NA, K	9	5 (12, 20, 25, 41, 43)	0.9
4	OXA, VA, P, OTC, S, SXT, AMP, NA	8	2 (4, 34)	0.8
5	OXA, VA, OTC, S, SXT, AMP, NA	7	4 (2, 22, 23, 44)	0.7
6	OXA, VA, P, OTC, S, SXT, AMP	7	2 (17, 21)	0.7
7	VA, P, OTC, S, SXT, AMP, NA	7	1 (13)	0.7
8	OTC, S, SXT, AMC, AMP, NA	6	3 (5, 26, 45)	0.6
9	S, SXT, AMC, AMP, NA, K	6	4 (6, 10, 28, 29)	0.6
10	VA, P, OTC, S, SXT, AMC	6	5 (7, 9, 27, 49, 50)	0.6
11	OXA, VA, P, OTC, S, K	6	3 (35, 36, 39)	0.6
12	OXA, VA, P, AMP, NA	5	1 (14)	0.5
13	OXA, VA, P, S, NA	5	4 (11, 30, 38, 46)	0.5
14	OXA, VA, OTC, S, NA	5	2 (31, 32)	0.5
15	P, S, NA, K	4	5 (15, 16, 40, 47, 48)	0.4
16	SXT, AMC, AMP, NA	4	1 (37)	0.4

*OXA* oxacillin, *VA* vancomycin, *P* penicillin G, *OTC* oxytetracycline, *S* streptomycin, *SXT* sulphamethazole/trimethoprim, *AMC* amoxicillin/clavulanic acid, *AMP* ampicillin, *NA* nalidixic acid, *K* kanamycin.

### Transmission electron microscope (TEM) characterization of isolated bacteriophage against multidrug resistance MRSA

**Figure 1:**
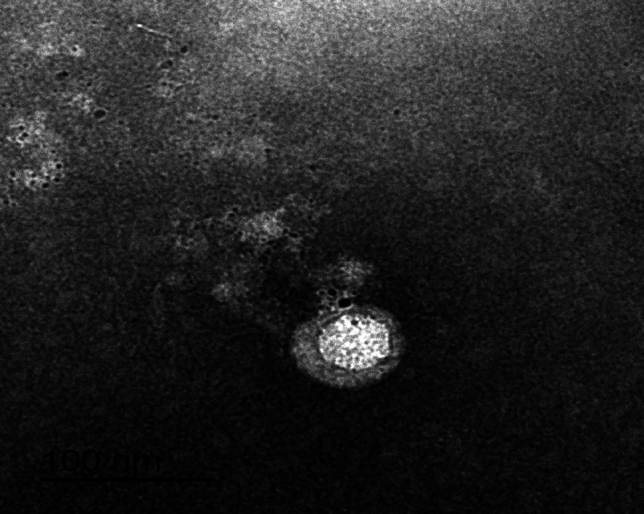
Transmission electron microscopy photograph of isolated MRSA phages at 500 nm of magnification value.

The phenotypic characterization of the isolated MRSA phages was detected by using TME (Fig. 1). Isolated MRSA phages belong to *Drexlerviridae* family virus according to new version of classifiction as it had an icosahedral-isometric head without tail.

### One-step growth curve

**Figure 2:**
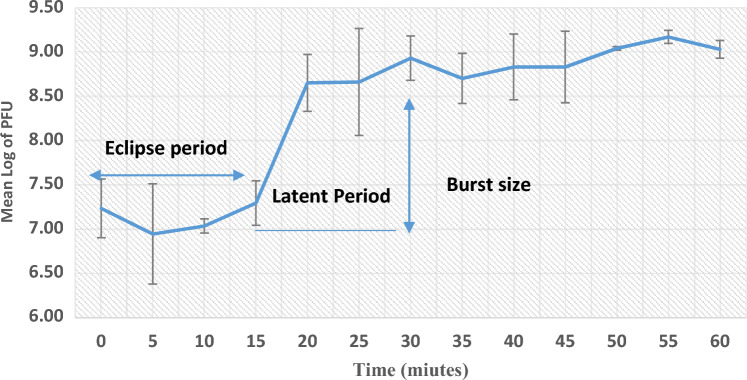
One step growth curve of MRSA phage. Eclipse, latent period and burst size of MRSA phage.

A one-step growth curve of MRSA phage propagated on multidrug resistant MRSA strain*.* 92% of the phage particles had adsorbed to the bacterial host cell after 15 min of incubation. Latent and eclipse periods were detected at 30 and 15 min, respectively. The burst size of MRSA phage was 32 PFU per infected cell (Fig. 2).

### Transmission Electron Microscope (TEM) characterization of chitosan nanoparticles (CS-NPs)

**Figure 3:**
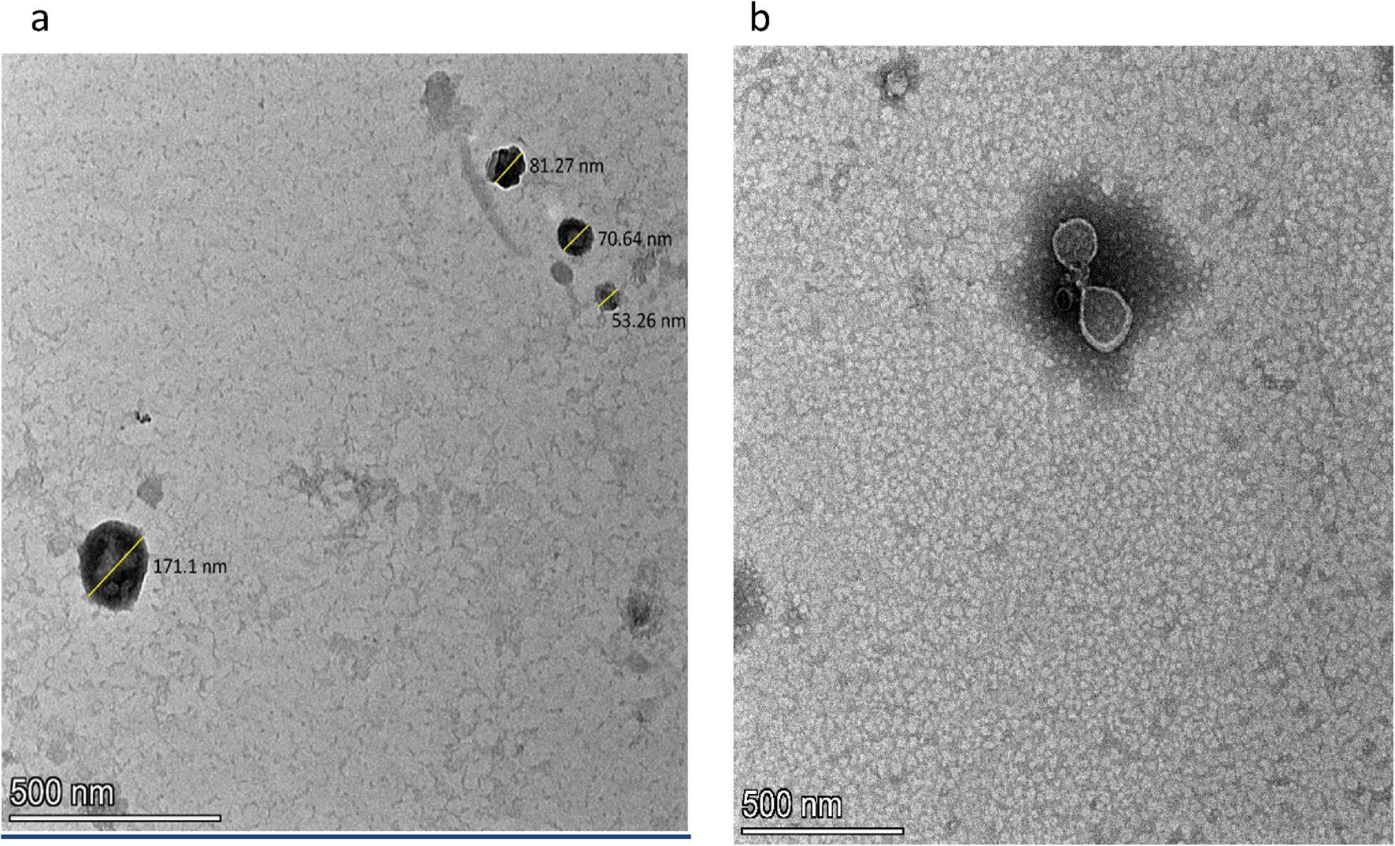
TEM analysis at 500 nm of magnification value of chitosan nanoparticles (**a**) and encapsulation of phage into chitosan nanoparticles (**b**).

As shown in Fig. 3a, TEM analysis of nano chitosan revealed the particles of chitosan in the range of nanoscale. Thus, it was found that some nanoparticles are varied in the size ranges, for example, 53.26, 70.64, 81.27, and 171.10 nm using magnification value of 500 nm. It was evident for the formulation of the nanoparticles in the solution. Besides, regarding the shapes of the nanoparticles we noted that the nanoparticles have no definite regular shapes, particularly tetragonal or needle shapes providing a large space of surface area. In addition, the nanoparticle did not tend to be aggregated in the solution, but these nanoparticles might have accumulated on the surface of large particles. The biological characteristics of chitosan nanoparticles should be improved in most cases as the efficiency of these nanoparticles was increased by increased surface area and aggregation factor.

### The encapsulation of phage into chitosan nanoparticles

The goal was to achieve a successful defense of bacteriophage against gastrointestinal digestive enzymes in the intestinal tract of the bird by the encapsulation of phage into chitosan nanoparticles or chitosan-phage-loaded nanoparticles which approved by TEM analysis of the encapsulation of phage into chitosan nanoparticles at 500 nm of magnification value Fig. 3b.

The phage is inactivated after oral consumption due to the effect of stomach acid and enzymes. The results of this investigation demonstrated that phage encapsulation in CS-NP as a carrier can prevent the phage from being enzymatically damaged and increasing its ability to resist acidic of stomach (pH 1.3).

### pH and thermal stability

**Figure 4:**
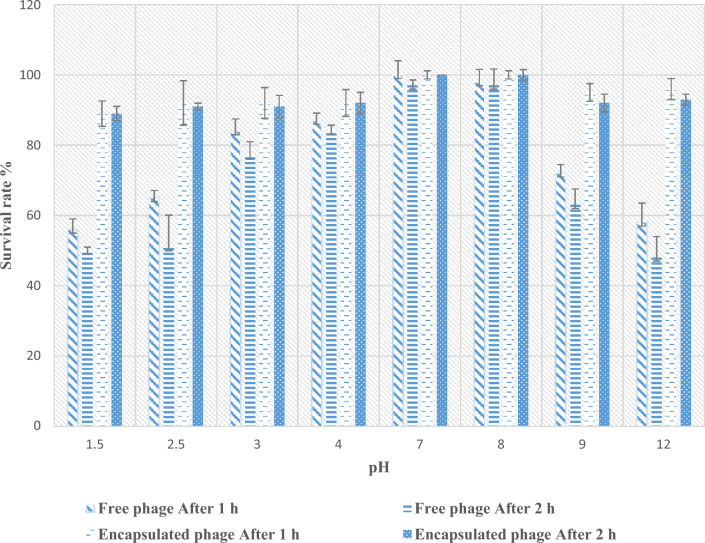
The survival rate of isolated MRSA phage and encapsulated phage with CS-NPs at different temperature degrees were shown at 2 h and 4 h of incubation from the triplicate experiments.

The stabilities of encapsulated MRSA phage with CS-NPs and free MRSA phage at different pH and temperature degrees were detected by determining the changes in the survival rate based on the number of PFU (Supplementary Information). Figure 4 showed that the growth of MRSA phage showed no obvious change after 1 h with pH 7.0–8.0 at which the survival rate was 100% and 98%, respectively (Fig. 4). The survival rate of MRSA phage after 1 h and 2 h incubation decreased with increasing alkalinity and acidity. The survival rate at pH 9.0 and 12.0 was 72% and 58%, respectively. Also, after 1 h at pH 1.5 and 2.5 the lowest survival rate of MRSA phage was detected to be 56% and 65%, respectively and after 2 h the survival rate was 50% and 51% (Fig. 4). Meanwhile, the lowest survival rate of CS-NPs encapsulated phage was 89% after 1 h and 2 h at pH 1.5 and was 91% to 100% at others examined pH levels (Fig. 4).

**Figure 5:**
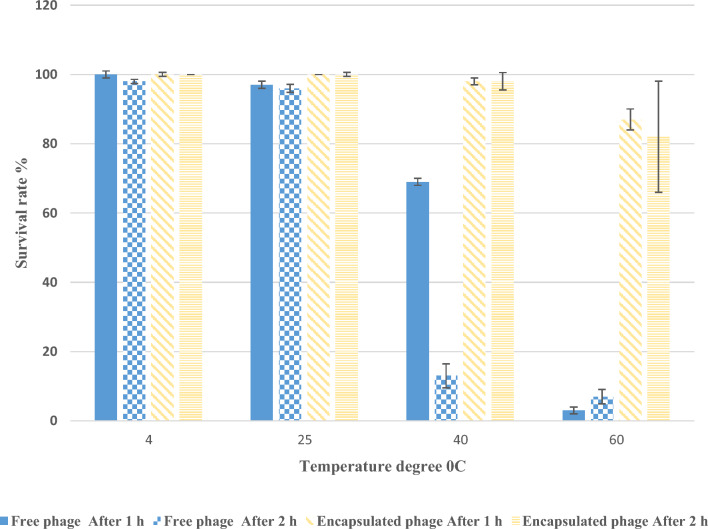
The survival rate of isolated free MRSA phage and encapsulated phage with CS-NPs at different pH were shown at 2 h and 4 h of incubation from the triplicate experiments.

The thermal stability of the MRSA phage was found at pH 7.0. The highest survival rate (100% to 98%) of MRSA phage was after incubation at 4 °C for 1 h and 2 h (Fig. 5). MRSA phage relatively stable at 25 °C for 1 h and 2 h at which the recovery of phage was 97% and 96%, respectively. Meanwhile, MRSA phage was sensitive to higher temperatures at which 3% and 7% of phage only survive after 1 h and 2 h of incubation at 60 °C, respectively.

### Host cell lysis (time-kill curve)

**Figure 6:**
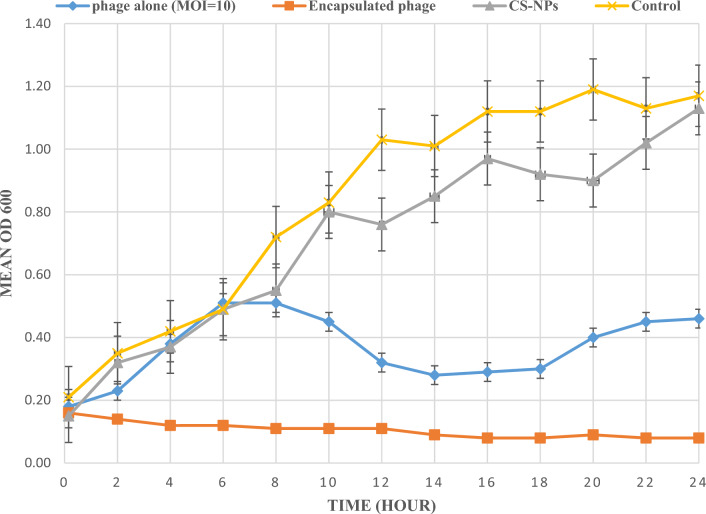
The time–killing curve of Multidrug resistant MRSA strain with MRSA phage alone, with CS-NPs alone and with CS-NPs encapsulated phage.

The in vitro efficacy of isolated MRSA phage alone, CS-NPs alone and the encapsulated MRSA phage with chitosan against multidrug-resistant MRSA was measured and represented in a time-kill curve to determine the differences in the optical density (OD_600_) changes. MRSA phage alone at an MOI of 10 had the ability to inhibit bacterial growth after 8 h of treatment by 0.38 OD_600_ compared with the control, and this effect persisted only for 8 h (Fig. 6). The data showed that CS-NPs alone was able to decrease the OD_600_ to 0.76 at 12 h, which persisted for 2 h of treatment then the bacterial growth begin to increase. Meanwhile, the bacterial growth in the presence of encapsulated MRSA phage with CS-NPs continued to decrease for 16 h then remained constant until 24 h from the beginning of the experiment (Fig. 6). We noticed that reactivation and regrowth of bacteria cells occurred with free phage, but it was defeated with encapsulated phage with CS-NPs. Overall, the encapsulated MRSA phage (MOI of 10) with CS-NPs had a significant inhibitory effect in comparing to the phage alone or even with CS-NPs alone.

## Discussion

MRSA was isolated with low prevalence from broiler chickens (31.8%; 159 out of 500 samples) and from the environmental samples of broiler chicken farms (27%; 27 out of 100 samples). Lee^30^ discovered a low incidence (17%) in broiler. Meanwhile, slightly higher prevalence of MRSA was recorded in broiler chickens by Bounar-Kechih et al.^31^ and Benrabia^32^ with 50% and 48.4%, respectively. Information regarding MRSA in the water, feed, and litter of chicken farms is not currently available. Meanwhile, MRSA was found in our study's environmental sample which was primarily found in water and litter with 48% and 28%, respectively and followed by worker hand swaps (20%) and feed (12%). Our findings may indicate that litter and water were the principal sources of MRSA to broiler chicken in the examined farms.

The emergence of antibiotic resistance is one of the increasingly critical global health problems^33^. Using the antimicrobial susceptibility test's recorded results as a reference, higher resistance was recorded to Streptomycin (S) (96%) followed by penicillin G (P) and sulfamethoxazole/trimethoprim (SXT) (72% for each), Oxytetracycline (OTC) and Nalidixic acid (70%). Our results diverge from earlier research by Abd-Allah et al.^34^ that recorded the susceptibility patterns and found that the majority of isolates were resistant to nearly all of the examined drugs, with the exception of vancomycin and linezolid. Also, Benrabia et al.^32^ reported resistance of MRSA isolates for tetracycline (82.5%), erythromycin (70.6%), clindamycin (68.6%), and ciprofloxacin (50%). Vancomycin (100%), mupirocin and rifampicin (99.2%), then chloramphenicol (82.3%) and gentamicin (76%), were almost universally effective against isolates.

Multidrug resistances were noted in furthermost isolated MRSA strains with MARI ranged from 1 to 0.4. Globally, antimicrobial resistance poses a serious threat to both human and animal health. Broiler-isolated MRSA isolates were cross-resistance to many other antibiotic families besides *β*-lactam antibiotics and were more resistant to single or multiple antibiotic combinations. Previous research provides good support for this antibiotic resistance data, particularly for poultry^35^. They could be harmful to human health in this regard.

The findings of this study demonstrate the need for additional control initiatives to reduce MRSA contamination and spread in the poultry sector, which will ultimately affect people. Therefore, the second part of our study aims to find biological replacement to the use of antibiotics inside the poultry farms. This replacement represents in the isolation of bacteriophage against MRSA strains and evaluating its inhibitory effect.

The morphology seen under TEM, which is crucial for phage characterization, was used to categorize isolated phages^36^. The head and tail of the phage's properties served as the basis for classification. The current research represented that the isolated MRSA phage was a member of the *Drexlerviridae* (*Podoviridae;* in old version of classification) family viruses which had a very short tail. The most infective phage and one with the shortest tail was LMP3. The length of the tail offers information on the stability and resistance of the phage in the wild; short and non-tailed phages are extra resistant, whereas lengthy tails are more easily killed, which reduces the phage's infectivity^37^. Similar MRSA-specific phages from the *Podoviridae* family were recovered from farm animals in a related study, and they were viewed using electron microscopy to demonstrate the potential for employing lytic bacteriophages against MRSA strains^38^. In additional research, 2 novel phages that were isolated from a farmyard and placed in the *Siphoviridae* family were found to have lytic activity against MRSA^4,39^. According to certain studies, *Siphoviridae* members make up the great majority of *S. aureus* phages^40,41^.

According to the distinctive characteristics of the isolated MRSA phage, the burst size of 32 PFU per infected cell, the latent duration of 30 min, and the adsorption rate (92%) were somewhat lower than those reported for phages by others^42,43^. A new generation of phage would start to spread within 30 min, according to the latent period, which exhibited that the amount of time taken to reproduce the virus inside the host is quite low. This study's progeny yield was within permissible limits and produced the high concentrations required for phage therapy. A benefit for antibacterial activity was also the phage's fast adsorptive attachment to the host bacterium. The isolated phage could be treated because of all of these characteristics.

Several physicochemical variables, including temperature and pH, have an impact on the free phage infectivity^22,44^. As a result, several physicochemical situations were used to test the isolated MRSA-phage. Along the pH range from 1.5 to 12, survival rate of MRSA phage was decreased to be less than 50%. Our findings were in contrast with that found by Abdallah et al.^21^; who detected that when MRSA phage was introduced in an environment which is either one strongly acidic (pH = 1, 2) or excessively alkaline (pH = 12, 13), the lytic spot vanished. This trend resembles that described in a previous study^45^, which found that the isolated *Sipho*-virus was unaffected by pH levels between 3 and 12. The bacteriophage vB_SauM_CP9 was rendered inactive beyond the pH levels between 4 and 9 in another investigation conducted by Abdallah et al.^21^. On another hand, the CS-NPs encapsulated phage showed no obvious changes in the survival rate compared with free phage. The encapsulation process plays a very important role in the protection of phages against adverse environmental conditions such as acidity and oxidation^13^.

All of the examined temperatures, with the exception of 60 °C, where the isolated MRSA phage entirely lost its lytic activity, saw the lytic activity of the phage continue. Thus, for shelf storage and general toleration of hot weather, 60 °C was regarded to be its thermal inactivation point even after just 1 h. This finding was in line with the comparable thermal stability research carried out by González-Menéndez et al.^44^. They found that after being a 90-min exposure to a temperature of 60 °C, the phage examined lost its ability to infect^46^. This level of heat stability is also higher than that of other well-known *S. aureus* bacteriophages reported in earlier research^47^, that completely lost their lytic activity above 50 °C. Meanwhile, our results revealed thermal stability of CS-NPs encapsulated phage even at 60 °C at which the survival rate was 82% after 2 h of incubation.

Our results refer to that the survival rate of MRSA phage was affected by different Ph and temperature degrees which necessitate its encapsulation inside CS-NPs to maintain its survival rate and increasing its resistant to the environmental conditions from one side and increasing its penetration to the bacterial cells from another side.

The analysis results regarding the study of surface morphology of prepared CS-NPs were consistent with the literature reported by Otunola et al*.*^27^. The pattern of release was most likely caused by the chitosan matrix swelling, which encourages the protonation of their amino groups under the acidic state, causing the phage to disintegrate and gradually diffuse from the nano-polymers matrix^12^. These results are consistent with the research by^47^, who showed that CS-NPs could protect phage from degradation by the enzyme pepsin *in-vitro* experimentation. It was also shown in gel electrophoresis those phages enclosed in chitosan nanoparticles are protected from the destruction by acidic environments and enzymes, whereas naked bacteriophages are destroyed in such circumstances.

Lytic activity of MRSA phage alone, CS-NPs alone and encapsulated phage with CS-NPs were evaluated against multidrug resistant MRSA strain. Our results revealed to the encapsulated MRSA phage with CS-NPs had a significant inhibitory effect and the bacterial growth continued to decrease for 16 h then remaining steady constant until the end of the experiment. The most crucial findings were that bacterial persistence took place when the phage and CS-NPS were used alone, but it was defeated when the phage was encapsulated with CS-NPs. Thus, significant inhibitory influence was observed in comparison to using the phage alone or even with using CS-NPs alone.

## Conclusion

The prevalence of MRSA was 31.8% at the level of birds, and 27% in the environmental samples. All isolates (100%) had MDR to four or more antibiotics, with MARI of 0.4–1. MRSA strains in this study demonstrated 16 different antibiotic resistant patterns. CS-NPs act as a protective barrier for MRSA phage before oral administration to farm animals and provide acid and thermal stability for the control of multidrug resistant MRSA in broiler chicken farms. Further research should be applied to study the effect of encapsulated MRSA phage application *in-vivo* on the control of multidrug resistant MRSA strain.

### Supplementary Information

Supplementary Information.

## Data Availability

All data generated or analyzed during this study are included in this published article and its supplementary information files.
